# Application of Doehlert design in optimizing the solid-state hydrogenogenic stage augmented with biomass fly ash in a two-stage biohythane production process

**DOI:** 10.1007/s00449-023-02873-6

**Published:** 2023-04-14

**Authors:** Seyedeh Azadeh Alavi-Borazjani, Helena Gil Martins de Faria Gomes, Luís António da Cruz Tarelho, Maria Isabel Capela

**Affiliations:** Department of Environment and Planning/Centre for Environmental and Marine Studies (CESAM), University of Aveiro, Campus Universitário de Santiago, 3810-193 Aveiro, Portugal

**Keywords:** Biohythane, Biohydrogen, Two-stage AD, Solid-state AD, Biomass ash, Doehlert design

## Abstract

This study aimed to optimize the solid-state hydrogenogenic stage supplemented with biomass fly ash in a two-stage anaerobic digestion (AD) process for biohythane production from the organic fraction of municipal solid waste (OFMSW). Doehlert’s experimental design was used to obtain the optimal set of two investigated variables, namely total solids (TS) content and biomass fly ash dosage in the defined ranges of 0–20 g/L and 20–40%, respectively. Applying the optimal conditions of TS content (29.1%) and fly ash dosage (19.2 g/L) in the first stage led not only to a total H_2_ yield of 95 mL/gVS_added_, which was very close to the maximum H_2_ yield predicted by the developed model (97 mL/gVS_added_), but also to a high CH_4_ yield of 400 mL/gVS_added_ (76% of the theoretical CH_4_ yield). Moreover, the biohythane obtained from the optimized two-stage process met the standards of a biohythane fuel with an H_2_ content of 19% v/v.

## Introduction

The two-stage anaerobic digestion (AD) process has attracted considerable attention in recent years for the sequential production of hydrogen (H_2_) and methane (CH_4_) as key components of a clean energy carrier called biohythane. Unlike other hythane production methods, this technology does not rely on fossil fuel consumption and can also ensure an adequate H_2_/CH_4_ ratio in the hythane by allowing for the adjustment of key parameters that affect the process. In addition, the production of hythane through the two-stage biological process can provide a sustainable solution to deal with the huge amount of biowaste produced in the world [1].

Based on the total solids (TS) content of the substrates used, AD processes can be operated in two different modes: wet or liquid-state AD (when the TS content is less than 15%) and dry or solid-state AD (when the TS content exceeds 15%) [2]. Dry AD is claimed to be superior to wet AD for several reasons, including (i) allowing more quantity of feedstock to be loaded into a smaller volume of the bioreactor; (ii) requiring less water as well as less input energy for heating and mixing the digester contents, and (iii) facilitating the management of the digested materials remaining after the process [3, 4]. However, despite the advantages of solid-state AD, the feasibility of using high TS contents in the fermentative H_2_ production process has been little investigated. In fact, very high levels of TS content in anaerobic fermentation would inhibit the process and reduce H_2_ production, mainly due to poor energy/mass transfer and excessive accumulation of organic metabolites [5]. Therefore, adopting a suitable strategy seems to be necessary to overcome this constraint and for any full-scale application of the solid-state fermentation process to produce H_2_ either as a single product or as a component of biohythane.

Recently, the use of biomass combustion ash as an additive has shown promising results in H_2_ production from organic waste, in an individual fermentation process [6] or in a two-stage process for biohythane production [7]. Nevertheless, previous studies were conducted under wet conditions and the effect of biomass-derived ash on H_2_ production from solid-state fermentation of organic matter is still unknown. Given that biomass fly ashes are usually characterized by a high buffering (acid-neutralizing) capacity [8], it seems that augmenting H_2_-producing reactors with such abundant inorganic waste can be an effective and economic strategy to deal with the over-acidification phenomenon that usually occurs at high levels of TS content. Therefore, in order to take advantage of solid-state fermentation and also achieve a desirable biohythane composition, the present study attempts to optimize H_2_ production at relatively high TS contents by adding biomass combustion fly ash to the first stage of a dual-stage AD process for biohythane production from the organic fraction of municipal solid waste (OFMSW).

The application of the uniform shell design, proposed by Doehlert in 1970, gained strength in the design of experiments and in the process optimization due to its advantages over classical methods. For example, compared to frequently used methods such as central composite or Box-Behnken, Doehlert design requires fewer experimental trials while being more efficient [9]. In addition, it allows the selection of a different number of levels for each variable, as well as adding new factors without changing the design quality. The ability to provide a uniform distribution of experimental points in the experimental domain and the usefulness of response interpolation are other advantages of the Doehlert design, which makes it stand out from other conventional techniques for experimental design and optimization [10]. Accordingly, the Doehlert technique is used in the H_2_ production phase of this study to quickly find the optimal conditions for the evaluated variables (i.e., TS content and fly ash dosage) in the specified range by performing a smaller number of experiments.

## Materials and methods

### Substrate, inoculum and biomass fly ash

A simulated OFMSW containing 95% food waste and 5% paper, on a wet weight basis, was used as the main substrate in this study. The food waste fraction of the simulated substrate consisted of 78% fruits and vegetables, 8% cooked meat and fish, 6% bread and bakery, 5% boiled pasta and rice, 2% dairy (cheese), and 1% confectionery and snacks (biscuits). This composition was formulated and modified based on a composition reported by VALORGAS [11] for European countries. Before use, the substrate was minced with an electric meat grinder to a particle size of less than 2 mm and kept in a freezer (− 20 °C) to prevent spoilage. The main characteristics of the prepared substrate were as follows: total solids (TS): 20.4 ± 1.94% wt., volatile solids (VS): 19.7 ± 2.56% wt., carbon (C): 45.7 ± 0.33% wt., oxygen (O): 39.7 ± 0.52% wt., nitrogen (N): 2.3 ± 0.14% wt., hydrogen (H): 7.1 ± 0.4% wt., and C/N ratio: 19.9.

The inoculum used in this work was an anaerobically digested sludge from a wastewater treatment plant in Aveiro, Portugal, characterized by a pH of 7.1 ± 0.15, TS of 1.9 ± 0.74% wt., and VS of 1.5 ± 0.68% wt. In order to inactivate methanogens and enrich spore-forming bacteria, the inoculum was subjected to an acid-shock treatment with 1 N HCl up to pH = 3 [12] and then cultivated in a medium as described by Zhang and Wang [13] at 37 °C under anaerobic conditions, 24 h before use in H_2_ production assays. As for the CH_4_ production stage, no inoculum treatment was performed.

The fly ash used in this study was obtained from the combustion of residual forest biomass, mainly composed of *Eucalyptus globulus* bark, in an industrial bubbling fluidized bed combustor located in Portugal. The basic characteristics of biomass fly ash are presented in Table 1.

**Table 1:** Basic characteristics of the biomass fly ash used in this study

Characteristics	Values
pH	13.5 ± 0.02
Loss on ignition at 1100 °C (%)	23.9 ± 1.84
*Chemical composition as oxides (%, dry weight basis)*	
Al_2_O_3_	3.0 ± 2.64
CaO	34.2 ± 1.55
Fe_2_O_3_	1.9 ± 1.03
K_2_O	7.5 ± 1.91
MgO	3.3 ± 2.52
MnO	1.2 ± 1.75
Na_2_O	4.2 ± 1.31
P_2_O_5_	0.9 ± 1.26
SO_3_	1.0 ± 2.03
SiO_2_	9.7 ± 1.02
TiO_2_	0.2 ± 1.78

### Experimental design and optimization

A Doehlert matrix was used to design the experiments and optimize the solid-state H_2_ production by considering two influential variables: substrate TS content and biomass fly ash dosage. The range of TS content and fly ash dosage investigated for H_2_ production, which were defined based on preliminary experiments (unpublished results), were 20˗40% (at intervals of 5) and 0˗20 g/L (at intervals of 10), respectively. The investigated response variable was the total H_2_ yield with the objective of maximization.

For the relevant calculations, the two independent variables under study were converted into coded variables according to the following equation [14]:

1 $${X}_{i} = \frac{{U}_{i}-{\overline{U} }_{i}}{\Delta {U}_{i}}$$

where ${X}_{i}$ is the coded value of the *i*^*th*^ variable; ${U}_{i}$ is the actual value; ${\overline{U} }_{i}$ is the value at the center of the study domain; and $\Delta {U}_{i}$ is the value of the step variation between the low level (˗1) and the high level (+ 1).

Nine experimental runs were planned to optimize H_2_ production in the present study with two independent variables and three central point experiments to estimate the experimental error using Eq. (2) [15]:

2 $$N={k}^{2}+k+{C}_{0}$$

where $N$ is the number of required experimental runs; $k$ is the number of factors (independent variables) taken into account; and ${C}_{0}$ is the number of experiments performed at the center-point.

The Doehlert experimental design with the actual and coded values of each factor is shown in Table 2.

**Table 2:** Doehlert experimental design with coded and actual values of the factors

Experiments	Codded values		Actual values	
	A	B	TS content (%)	Fly ash dosage (g/L)
H1	1	0	40	10
H2	0.5	0.866	35	20
H3	− 1	0	20	10
H4	− 0.5	− 0.866	25	0
H5	0.5	− 0.866	35	0
H6	− 0.5	0.866	25	20
H7 (CP)	0	0	30	10
H8 (CP)	0	0	30	10
H9 (CP)	0	0	30	10

Experiments H7, H8, and H9 are triplicates at the center point

Multiple regression analysis was performed on the experimental data and fitted to a second-order polynomial model according to the following general equation [16]:

3 $$R = {\beta }_{0}+\sum_{i=1}^{n}{\beta }_{i}{X}_{i}+\sum_{i=1}^{n}{\beta }_{ii}{X}_{i}^{2}+\sum_{i<j}^{n}{\beta }_{ij}{X}_{i}{X}_{j}$$

where $R$ is the response variable; ${\beta }_{0}$ is the constant term; ${\beta }_{i}$, ${\beta }_{ii}$, and ${\beta }_{ij}$ are regression coefficients for linear effects, squared effects, and interaction effects, respectively; and ${X}_{i}$ and ${X}_{j}$ are the independent variables (labelled as A and B here).

The obtained polynomial model subjected to a second partial derivative test to investigate whether the critical point of the function is a local maximum, minimum, or saddle point. This was done by calculating the determinant of the Hessian matrix for a function of two variables (A and B) according to Eq. (4) [17]:

4 $$D = \left[\begin{array}{cc} \frac{{\partial }^{2}R}{{\partial A}^{2}}& \frac{{\partial }^{2}R}{\partial A\partial B}\\ \frac{{\partial }^{2}y}{\partial B\partial A}& \frac{{\partial }^{2}y}{{\partial B}^{2}}\end{array}\right] = \left( \frac{{\partial }^{2}R}{{\partial A}^{2}}\times \frac{{\partial }^{2}y}{{\partial B}^{2}}\right) - \left(\frac{{\partial }^{2}R}{\partial A\partial B} \times \frac{{\partial }^{2}y}{\partial B\partial A}\right)$$

The critical point ${(a}_{0}, {b}_{0})$ is a maximum if $D>0$ and ${\partial }^{2}R/{\partial A}^{2}<0$. Conversely, there is a local minimum point if $D<0$ and ${\partial }^{2}R/{\partial A}^{2}>0$. In addition, a saddle point exists on the surface if $D<0$ [18].

After checking the geometric nature of the critical point on the response surface, its coordinates were calculated as optimal conditions by solving the system of equations $\partial R/\partial A=0$ and $\partial R/\partial B=0$, which were obtained from the first derivative of the generated mathematical model in relation to each variable [19].

Minitab software (version 18) and Design-Expert software (version 13) were used to design experiments, data processing, and graphics in the optimization of solid-state H_2_ production phase.

### Experimental setup and procedure

Batch dry H_2_ production experiments were conducted in duplicates using 1-L glass vessels over a fermentation period of 96 h. At the beginning of each dark fermentation test, the bioreactors were loaded with certain amounts of OFMSW corresponding to the designated TS levels as well as with acid-shocked anaerobic digested sludge at a fixed substrate to inoculum mass ratio of 2 on a VS basis [20]. The fermentation reactors were then supplemented with biomass fly ash at concentrations ranging from 0 to 20 g/L and filled with distilled water to reach a working volume of 400 mL.

The second-stage of CH_4_ production was performed in duplicate in 1-L glass vessels with a working volume of 600 mL. For this stage, the spent media from the H_2_ production stage was used as substrate. The amounts of substrate and anaerobically digested sludge loaded in the CH_4_-generating reactor were adjusted to a substrate to inoculum mass ratio of about 4 based on VS concentration. The entire CH_4_ production phase lasted 22 d in batch operation mode.

Before starting each test, the bioreactors were purged with pure N_2_ gas for 3 min and carefully sealed to establish anaerobic conditions. A thermophilic (stage I)-mesophilic (stage II) configuration is recommended to be beneficial for improving the biogas efficiency and stability of the two-stage AD process [21]. Hence, the operating temperatures of the H_2_ and CH_4_ production reactors were maintained at 55 °C and 37 °C, respectively, by a thermostat-connected water bath. The volume of biogas and its composition were monitored at 24-h intervals and stirring was performed manually twice a day for 1 min during each experiment.

### Analytical methods and calculations

TS and VS content analysis was performed according to standard methods [22]. The pH levels were monitored with a Consort C861 multi-parameter analyser equipped with a pH probe (Consort SP10T). The elemental composition (CHON) of the substrate was determined using an organic elemental analyser (Thermo Scientific FLASH 2000). The water availability of the feedstock was measured with an AwTherm water activity meter (ROTRONIC HYGROMER^®^ IN-1). A PerkinElmer Clarus^®^ 480 gas chromatograph (GC) with the characteristics described elsewhere [6] was used to quantify the VFAs. The chemical composition of biomass fly ash, expressed as oxides, was determined by X-ray fluorescence spectrometry (XRF, Panalytical Axios spectrometer).

The volume of biogas produced at each time interval was measured by the water displacement method. The recorded values of biogas volume were adjusted to standard conditions of temperature and pressure (STP; 0 °C and 1 atm). The biogas composition was analysed with a micro-GC (Fusion^®^ Gas Analyzer-INFICON) equipped with two analytical capillary columns, one for CO_2_ analysis using argon as carrier gas and the other for analysis of O_2_, H_2_, N_2_, and CH_4_ with helium as the carrier gas. The temperatures of the injector and detector were set at 90 °C and 80 °C, respectively.

The cumulative H_2_ or CH_4_ volume (mL) was calculated according to Eq. (5) [23]:

5 $${V}_{C, t} = {V}_{C, t-1}+{C}_{C,t}\left({V}_{B,t}-{V}_{B, t-1}\right) +{ V}_{H}\left({C}_{C, t}-{C}_{C, t-1}\right)$$

where ${V}_{C, t}$ and ${V}_{C, t-1}$ are the cumulative volumes of H_2_ or CH_4_ (mL) at current ($t$) and previous ($t-1$) time intervals; ${C}_{C,t}$ and ${C}_{C, t-1}$ are the volumetric percentage of H_2_ or CH_4_ measured by the GC at current and previous time intervals; ${V}_{B,t}$ and ${V}_{B, t-1}$ are the volumes of biogas (mL) produced at current and previous time intervals; and ${V}_{H}$ is the bioreactor headspace volume (mL).

The cumulative H_2_ or CH_4_ yields, expressed as mL/gVS_added_, were calculated by dividing the cumulative H_2_ or CH_4_ volumes (mL) by the unit mass of substrate (based on VS) initially added to the hydrogenogenic bioreactors [24].

Theoretical CH_4_ yield (mL/gVS_added_) was estimated using Eq. (6) considering the elemental composition (C_a_H_b_O_c_N_d_) of the input feedstock [25]:

6 $$Theoretical \,\,C{H}_{4}\,\, yield = \frac{22.4\times \left(\frac{a}{2}+ \frac{b}{8} - \frac{c}{4 } - \frac{3d}{8}\right)}{12.017a+1.0079b+15.999c+14.0067d}$$

where *a*, *b*, *c*, and *d* are constants of elements C, H, O, and N, respectively, which are obtained by dividing the elemental analysis-based mass by the molar mass of each element.

H_2_ energy yields (${EY}_{{H}_{2}}$, kJ/gVS_added_) and CH_4_ energy yields (${EY}_{{CH}_{4}}$, kJ/gVS_added_), as well as energy yield from the whole two-stage AD (${EY}_{T}$, kJ/gVS_added_), were calculated according to Eqs. (7–9) [7]:

7 $${EY}_{{H}_{2}} ={ H}_{2}\,\, yield\times {HHV}_{{H}_{2}}$$

8 $${EY}_{{CH}_{4}} = {CH}_{4}\,\, yield\times {HHV}_{{CH}_{4}}$$

9 $${EY}_{T} ={ EY}_{{H}_{2}}+{EY}_{{CH}_{4}}$$

where ${HHV}_{{H}_{2}}$ is the higher heating value of H_2_ (12.7 MJ/Nm^3^); and ${HHV}_{{CH}_{4}}$ is the higher heating value of CH_4_ (39.8 MJ/Nm^3^).

The global VS removal efficiency (%) in the two-stage biohythane production process was obtained by summing the VS removal efficiencies in the H_2_ and CH_4_ production stages, which were calculated using Eqs. (10) and (11), respectively [26]:

10 $${RE}_{H }= \frac{{VS}_{HS}+{VS}_{HI}-{VS}_{HF}}{{VS}_{HS}+{VS}_{HI}}$$

11 $${RE}_{M} = \frac{{VS}_{MS}+{VS}_{MI}-{VS}_{MF}}{{VS}_{MS}+{VS}_{MI}}$$

where ${VS}_{HS}$ and ${VS}_{MS}$ represent the VS of the substrate (g/L) used in the H_2_ production stage (i.e., OFMSW) and in the CH_4_ production stage (i.e., the spent media from the first fermentation stage), respectively. ${VS}_{HI}$ and ${VS}_{MI}$ denote the VS of the inoculum (g/L) used for the H_2_ and CH_4_ production stages, respectively. ${VS}_{HF}$ and ${VS}_{MF}$ are the VS at the end of the H_2_ production stage and CH_4_ production stage (g/L), respectively.

## Results and discussion

### First stage of H_2_ production

#### Optimization tests

**Fig. 1:**
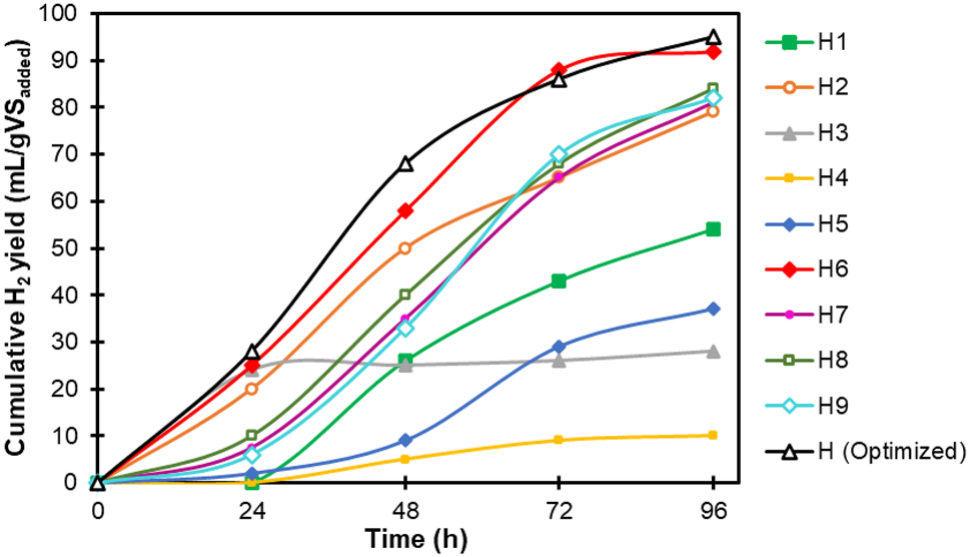
Cumulative H_2_ yields for 9 experiments designed and verification test (optimal conditions)

H_2_ production assays were performed in solid-state conditions as per Doehlert design using different substrate TS contents and biomass fly ash doses. The cumulative curves of H_2_ production over the fermentation period are illustrated in Fig. 1. As shown in Fig. 1, the H_2_ production performance followed different trends in the designed experiments, indicating that changes in TS content and fly ash dosage could significantly affect the fermentation process. The total H_2_ yield as the response variable varied between 10 and 92 mL/gVS_added_ with the maximum value for experiment H6 (TS content of 25% and fly ash dosage of 20 g/L) and the lowest value for experiment H4 (TS content of 25% and fly ash dosage of 0 g/L).

By performing a multiple regression analysis on the experimental data, the following polynomial second-order model in terms of uncoded (actual) values was derived:

12 $$R=-434.7+28.13 \,A+12.71 \,B-0.42 \,{A}^{2}- 0.18 \,{B}^{2} - 0.20 \,AB$$

where $R$ is the predicted H_2_ yield (mL/gVS_added_), and $A$ and $B$ are the TS content (%) and fly ash dosage (g/L), respectively.

**Fig. 2:**
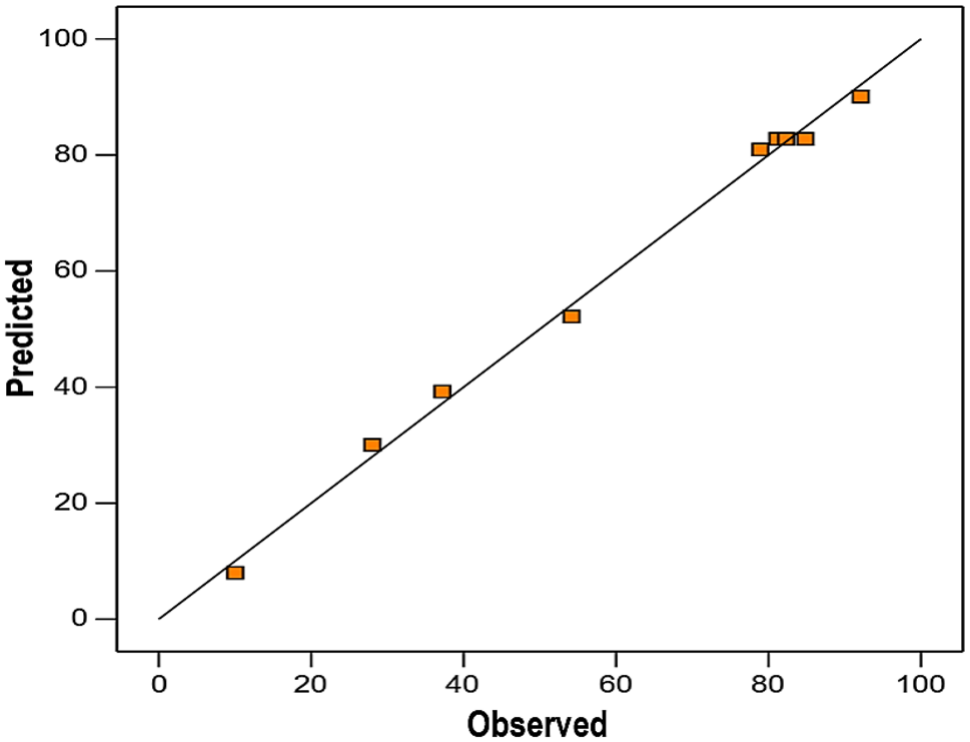
Parity plot of the observed and predicted values of the response (H_2_ yield)

The adequacy of the model was evaluated by an analysis of variance (ANOVA) for the response variable (see Table 3). Based on the ANOVA results, the calculated F-value for the regression model was 133 and the p-value was 0.001 (≤ 0.05), indicating that the model was highly significant. The lack-of-fit was also insignificant (p-value > 0.05), which implies that the model fitted well with the experimental data. The goodness-of-fit for the proposed model was further attested by the coefficient of determination (R^2^) which was 0.995. It means that 99.5% of the observed variation in the H_2_ yield was attributed to the independent factors of interest and only 0.5% of the response variability was not explained by the input variables. The value of adjusted R^2^ (0.988) was also high enough to support the model adequacy. Moreover, the parity plot depicted in Fig. 2 shows a good correlation between the experimental and predicted response values because the data points are concentrated adjacent to the diagonal line [27]. Therefore, it can be suggested that the proposed model was reliable for determining the optimal points of TS content and fly ash dosage, as well as for predicting the maximum H_2_ yield in this study.

**Table 3:** Results of analysis of variance (ANOVA) for the model

Source	DF^a^	SS^a^	MS^a^	F-Value	P-Value
Model	5	6983	1397	133	0.001
Linear	2	4196	2098	200	0.001
TS content	1	367	367	35	0.010
Fly ash dosage	1	3830	3830	364	0.000
Square	2	2380	1190	113	0.001
TS content*TS content	1	2084	2084	198	0.001
Fly ash dosage*Fly ash dosage	1	675	675	64	0.004
2-Way Interaction	1	407	407	39	0.008
TS content*Fly ash dosage	1	407	407	39	0.008
Error	3	31	10		
Lack-of-Fit	1	24	24	7	0.119^**b**^
Pure Error	2	7	3		
Total	8	7015			
R^2^ = 0.995 Adjusted R^2^ = 0.988					

^a^DF: Degrees of freedom; SS: Sum of squares; MS: Mean squares

^b^Insignificant at p-value ≤ 0.05

**Fig. 3:**
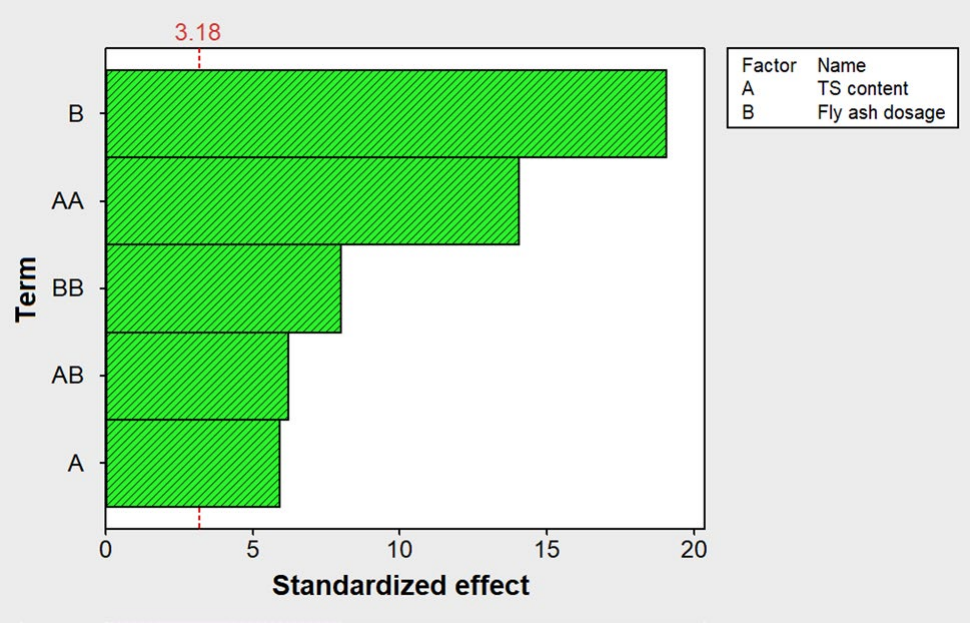
Pareto chart of the standardized effects for H_2_ yield

The standardized effects of the model terms are shown in Fig. 3 as a Pareto chart, in which the length of the bars is proportional to the magnitude (absolute values) of the estimated effects. The diagram also includes a reference line to show which effects are statistically significant. If the bar corresponding to each effect crosses the vertical reference line, it is considered significant [28]. According to the Pareto chart, all linear, quadratic, and interaction effects of the input variables were found to be significant at a confidence level of 95%. Among the current model terms, the greatest effect on H_2_ yield was related to the dose of fly ash in its linear form, followed by the quadratic effect of TS content.

**Fig. 4:**
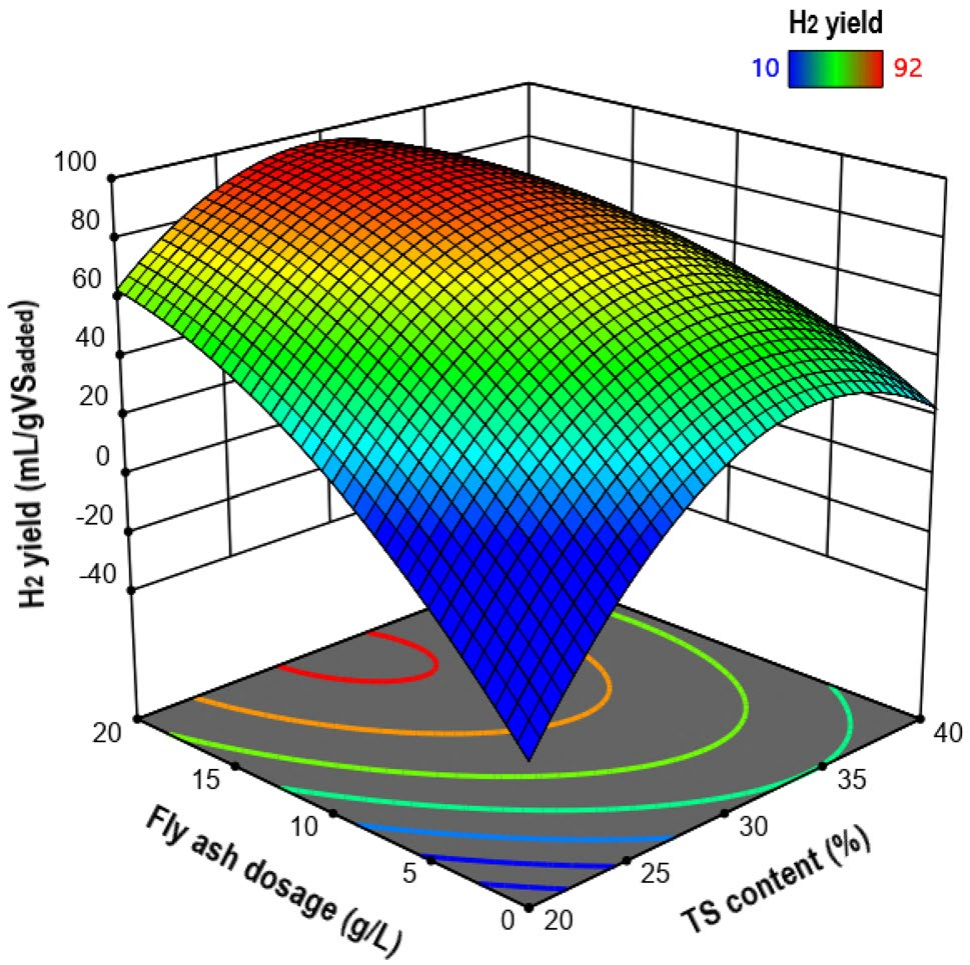
Response surface plot for H_2_ yield as a function of TS content and fly ash dosage

The three-dimensional response surface plot constructed from the regression model is depicted in Fig. 4. This graph visually shows that the optimal conditions for maximum H_2_ production are near the midpoint of the TS content and the highest dose of fly ash.

**Fig. 5:**
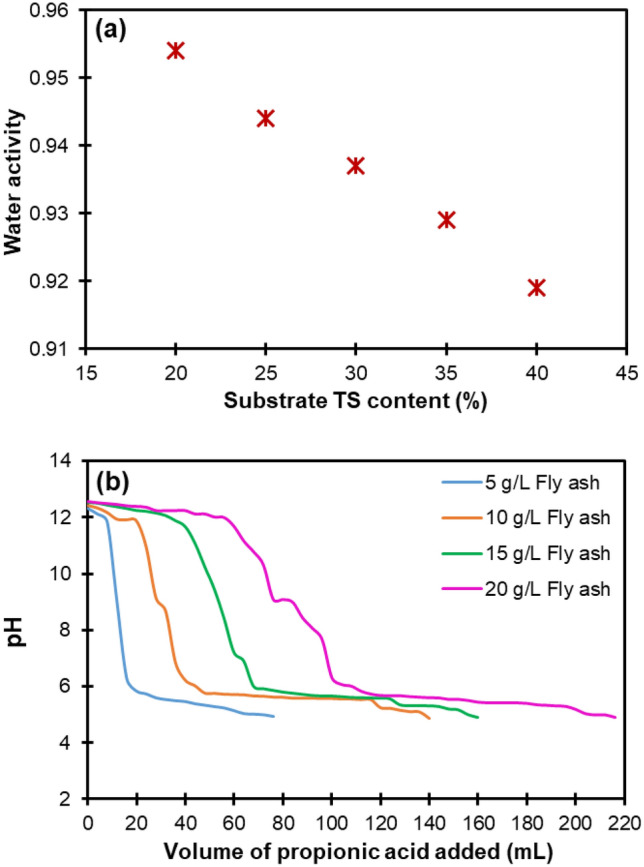
**a** Variation of water activity with TS contents of the substrate, and **b** titration curves for different concentrations of biomass fly ash suspended in distilled water with propionic acid

The positive sign of the coefficient calculated for the linear effect of the TS content in the polynomial model (Eq. (12)) shows the positive effect of this factor on H_2_ production. Nevertheless, according to the Pareto chart of the standardized effects (Fig. 3), the quadratic effect of TS content was very significant and higher than its linear effect, which resulted in a manifest curvature in the response surface. Hence, as can be seen in Fig. 4, increasing the TS content almost to the middle of the study domain caused the H_2_ yield to reach its maximum value, while further increasing the TS content decreased the H_2_ yield. It is known that the water activity in fermentation systems has a significant contribution to the transport of solutes and gases [29]. Also, it is believed that in a medium with low water activity, microorganisms need extra effort to grow because they must expend energy to maintain a high concentration of internal solutes to preserve water. As shown in Fig. 5a, the substrate water activity in this work decreased with increasing TS content. It is therefore likely that at TS contents above the optimal level, the metabolism of the respective fermentative bacteria was adversely affected by low water availability, which consequently led to a decrease in H_2_ production. In other words, the decrease in mass transfer and the increase in osmotic pressure resulting from the decrease in water availability can be the possible reason for the decrease in H_2_ production at TS contents beyond the optimal level.

Referring to Eq. (12), it can be seen that the linear effect of fly ash dosage on H_2_ production was also positive. The magnitude of this linear effect was the largest among all effects and, therefore, greater than the quadratic effect of fly ash dosage (see Fig. 3), which caused the response surface to show no apparent curvature due to this factor. The response surface (Fig. 4) demonstrates well that increasing the amount of fly ash from the lowest to the almost highest level in the investigated range led to a significant increment in the H_2_ yield as an output response. These results can be attributed to the increased buffering capacity of fly ash at higher doses, which in turn increases the possibility of overcoming excessive acidification as a serious impediment to the dark fermentative H_2_ production. Figure 5b illustrates the titration curves for different concentrations of biomass fly ash (5, 10, 15, and 20 g/L) suspended in distilled water with propionic acid as the most detrimental VFA in the biohydrogen formation process [30]. As shown in Fig. 5b, the higher the fly ash concentration, the more the pH drop was delayed, suggesting that increasing the dose of fly ash could boost the buffering (acid neutralizing) capacity and pH resistance in the reactive system. The higher buffering capacity of fly ash in higher doses is mainly due to the higher content of calcium-containing minerals [31].

**Fig. 6:**
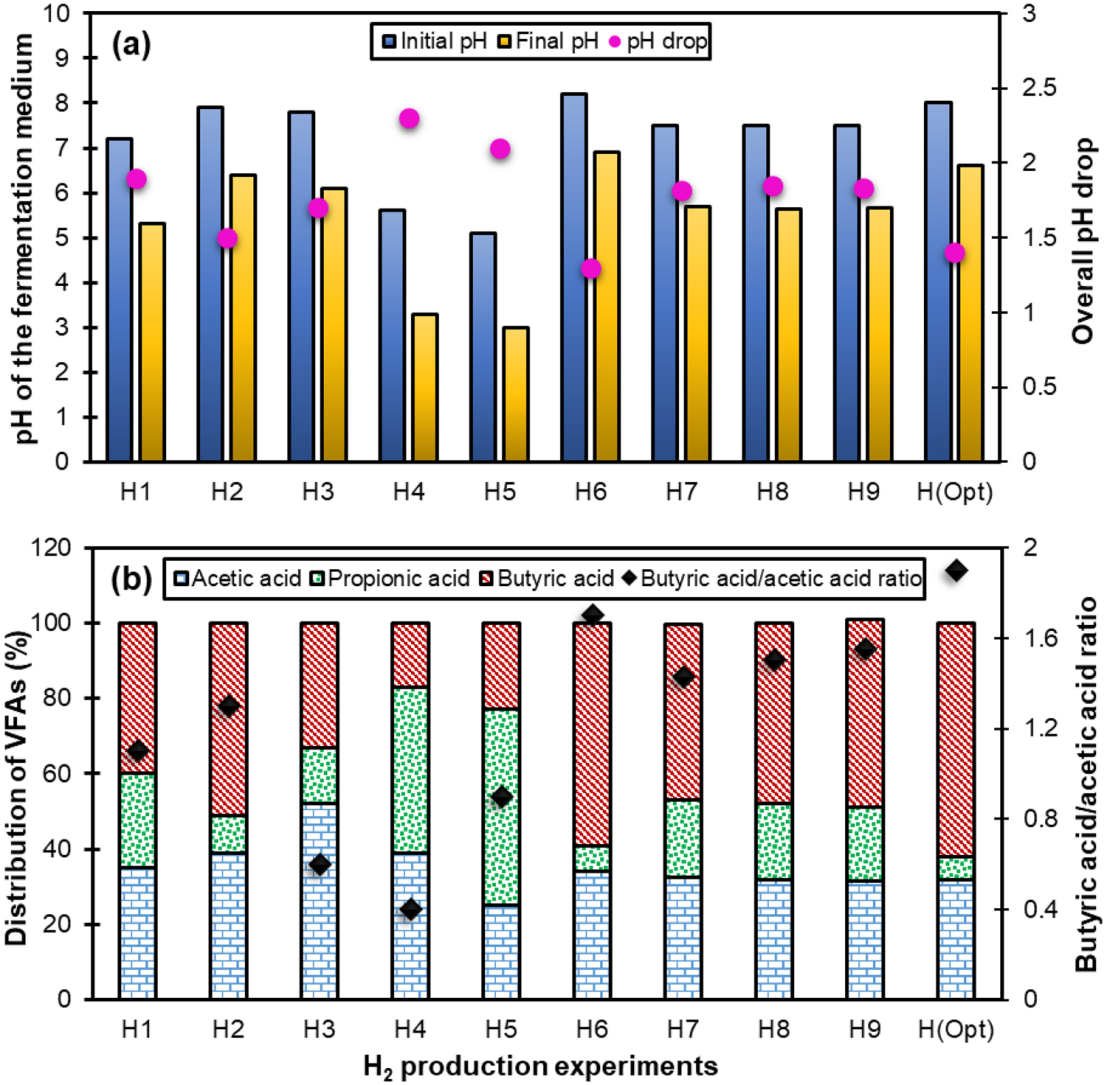
Comparison of (**a**) initial and final pH and total pH drop, and (**b**) distribution of VFAs and butyric acid/acetic acid ratio in H_2_ production assays

In order to further elucidate the buffering capacity of fly ash at different concentrations, the overall pH drop was evaluated in all designed batch experiments (see Fig. 6a). Regardless of the initial pH, a sharp drop in pH is usually expected at the end of the dark fermentative H_2_ production assays due to the accumulation of VFAs [32]. Apparently, in fermentation tests without adding fly ash (experiments H4 and H5), the initial and final pH difference was greater than in the other experiments supplemented with fly ash. Regarding the fly ash-added groups, the pH drop in the tests receiving 20 g/L of fly ash (experiments H2 and H6) was less than that in the trials using 10 g/L of fly ash (experiments H1, H3, H7, H8, and H9). These observations also indicate that the increase in fly ash dosage could prevent the excessive drop of pH and thus the occurrence of the over-acidification problem in the high-solid hydrogenogenic reactors.

At the end of each H_2_ production test, distribution of VFAs were also analyzed to identify the metabolic pathway governing the fermentation process. Acetic acid, butyric acid, and propionic acid were the only VFAs detected. These organic acids showed diverse distribution patterns under the tested conditions (see Fig. 6b). It is worth noting that the ratio of butyric acid to acetic acid, which is usually used as an indicator to evaluate H_2_ production performance, showed a direct relationship with the final H_2_ yield of the designed experiments. According to the literature, H_2_ production can be maximized if the fermentation process is predominantly oriented towards butyrate-acetate pathway [33]. In contrast, propionic acid-type fermentation is unfavorable for H_2_ production and should be avoided [30]. As can be seen in Fig. 6b, regardless of TS content, the propionic acid content in the total VFAs significantly decreased with the inclusion of fly ash and its increased concentration. Previous studies demonstrated that the addition of proper concentration of trace elements could successfully prevent the accumulation of VFAs even at high organic loading rates [34]. Some researchers even could restore acidified reactors by adding trace elements [35, 36]. Therefore, the reduction of propionic acid in experiments supplemented with fly ash can be ascribed to the improved synthesis of enzymes required for microbial metabolism with the help of trace elements released from fly ash. The final concentrations of several trace elements (Fe, Mo, Ni, Se, and W) detected in the H_2_ production experiments performed in this work, which have previously been used in propionic acid degradation studies, are presented in Table 4. The target trace elements showed different release behavior depending on their concentration in fly ash as well as their leaching characteristics. Regarding the effect of the trace elements investigated in this study on the degradation of propionic acid, it seems that Mo was the only effective element since its final concentration in different tested conditions followed a relatively inverse trend compared to that of propionic acid content. This is justifiable because Mo is present in a mononuclear form in the active site of formate dehydrogenase, which is a key enzyme in propionic acid degradation [37].

**Table 4:** Final concentrations of trace elements in H_2_ production experiments

Experiments	Trace element concentrations (mg/L)				
	Fe	Mo	Ni	Se	W
H1	62.3	12.5	2.6	2.9	3.8
H2	57.8	17.4	3.8	2.4	4.5
H3	41.6	14.5	2.1	1.8	3.1
H4	6.2	3.7	1.5	1.9	0.1
H5	8.1	3.6	1.8	2.1	0.2
H6	52.3	19.2	3.2	2	4.2
H7	66.9	13.6	2.2	1.7	3.7
H8	64	13.8	2.3	1.7	3.6
H9	65.1	13.8	2.2	1.6	3.5
H(Optimized)	51.6	21.6	3.5	2.7	4.4

**Fig. 7:**
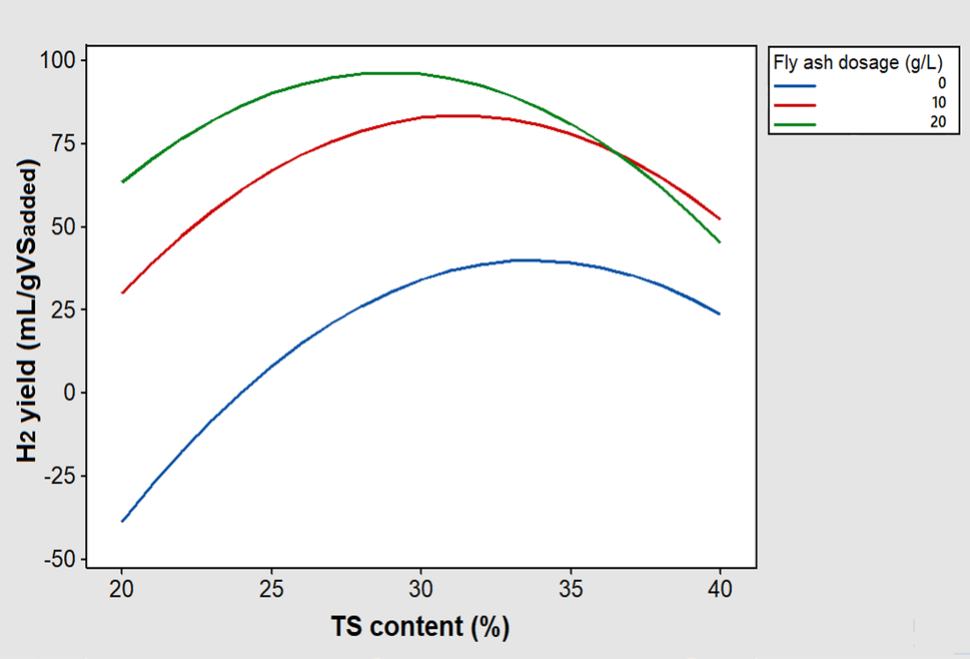
Interactive effect of TS content and fly ash dosage on H_2_ yield

According to the Pareto chart shown in Fig. 3, a statistically significant interaction was found between the TS content and fly ash dosage at a 95% confidence level. Although the magnitude of this effect was small, it does not seem rational to interpret the influence of the two studied variables on H_2_ production without considering their possible interaction. A graphical representation of the interaction between the TS content and fly ash dosage is shown in Fig. 7. This interaction plot shows how the relationship between TS content and H_2_ yield depends on the amount of fly ash added to the bioreactors. At TS contents between 20 and 35%, the amount of 20 g/L of fly ash was associated with the highest H_2_ yield, while when the TS content exceeded 35%, the addition of 10 g/L of fly ash had the greatest effect on H_2_ production. This result could be justifiable because, as previously described, an increase in the amount of fly ash can increase the buffering capacity and thus diminish the possibility of over-acidification in the solid-state H_2_ production process. However, at higher TS contents, adding a large amount of fly ash to the fermentation system can adversely affect process performance, most likely due to excessive dry matter loading.

The behavior of the response function at the critical point was evaluated by the second-order partial derivative test. Based on this test, it was found that $D=0.3>0$ and ${\partial }^{2}R/\partial {A}^{2}=-0.8<0$, thus confirming the existence of a maximum point on the response surface.

After solving the linear equations obtained from the first partial derivative of the response function (Eq. (12)) with respect to each of the studied variables, the values calculated for the TS content and fly ash dosage at the critical point were 29.1% and 19.2 g/L, respectively, indicating that the maximum point appears in the experimental domain. The values found for the critical point can be considered optimal conditions for the investigated variables when they represent the point of the function where the response (H_2_ yield) is maximized.

#### Verification test

The predicted optimal conditions for TS content (29.1%) and fly ash dosage (19.2 g/L) were subjected to a solid-state batch dark fermentation test in vitro, labelled as H(optimized), to confirm the reliability of the created model. As shown in Fig. 1, the final H_2_ yield under optimal conditions was 95 mL/gVS_added_, very close to the maximum H_2_ yield (97 mL/gVS_added_) estimated by the model with a relative error of 2.1%. It was also higher than the values obtained from the nine experiments designed by the Doehlert method to produce H_2_. To the best of the authors’ knowledge, in other solid-state batch fermentation experiments previously performed using organic solid wastes, optimal TS contents were lower than that found in this study. For example, Ghimire et al. (2018) carried out a series of dark fermentation experiments with food residues at different TS contents (10, 15, 20, 25, and 30%) and observed that the H_2_ production was stopped at TS contents above 15%, accompanied by lactic acid accumulation. In another work by Valdez-Vazquez and Poggi-Varaldo (2009), the highest H_2_ yield was obtained from OFMSW at a TS content of 20.9% in a tested TS range of 20.9% to 35.1%. Nevertheless, in the current work, the maximum H_2_ yield was achieved using a higher TS content (29.1%) without the fermentation system being exposed to excessive acidification. This may be due to the fact that by using the appropriate amount (19.2 g/L) of biomass fly ash, sufficient buffering capacity was provided to the system, thus the H_2_-producing reactor was able to withstand more TS load compared to previous studies.

As depicted in Fig. 6a, the pH value recorded at the beginning of the H_2_ production phase under optimal conditions was 8.0, which was consistent with previous studies [39, 40] in which the same initial pH maximized H_2_ production from the fermentation process of similar substrates. Furthermore, a relatively high final pH of 6.6 for this experiment indicated that the pH drop was very small, which further implies that the buffering capacity provided by fly ash most probably prevented a large decline in pH, despite the fact that the fermentation process took place under dry conditions with a high risk of excessive acidification. Also, the analysis of VFAs at the end of the hydrogenogenic experiment under optimal conditions showed that butyric acid and acetic acid were the main VFAs detected with distribution percentages of 62% and 32%, respectively. However, the concentration of propionic acid was very low, accounting for only 6% of the total VFAs (see Fig. 6b), indicating that a preferential metabolic pattern occurred during the H_2_ production process under optimal conditions of TS content and fly ash dosage.

Taken together, the above results suggest that the developed model has sufficient adequacy to predict the optimal points of TS content and fly ash for maximizing H_2_ production under dry fermentation conditions.

### Second stage of CH_4_ production

**Fig. 8:**
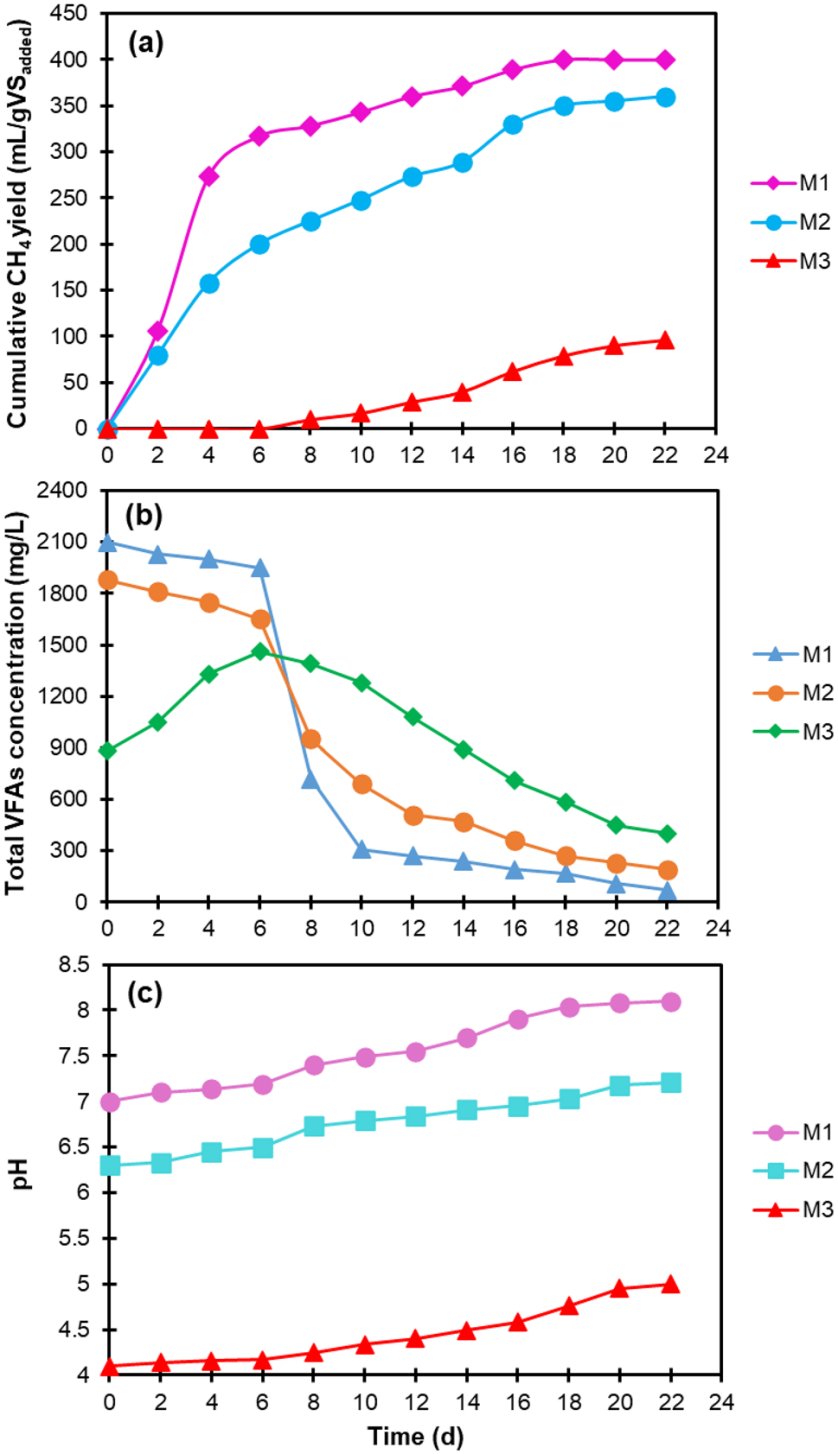
Variation of (**a**) cumulative CH_4_ yield, (**b**) total VFAs concentration, and (**c**) pH during the methanogenic stage

In the second stage, three experiments labelled as M1, M2, and M3 were carried out for CH_4_ production, in which the spent media from the fermentation experiments H(Optimized) with the highest H_2_ yield, H9 as a representative of the three experiments carried out in the central points, and H4 with the lowest H_2_ yield were used as substrates, respectively. As can be seen in Fig. 8a, the three CH_4_ production assays exhibited different performances at this stage. The cumulative CH_4_ yield at the end of the process for experiments M1, M2, and M3 were 400, 360, and 98 mL/gVS_added_, respectively. To the best of the authors' knowledge, the highest CH_4_ yield obtained in this work was higher than those obtained in other two-stage AD configurations consisting of a first solid-state acidogenic stage and a second-stage methanogenic digester using similar feedstocks [41–46]. On the other hand, the experimental CH_4_ yield obtained from experiment M1 was about 76% of the theoretical CH_4_ yield (526 mL/gVS_added_) calculated by Eq. (6), while those attained from experiments M2 and M3 were about 68% and 19% of the theoretical CH_4_ yield, respectively.

The variation of total VFAs during the methanogenic stage is illustrated in Fig. 8b. In the first 6 days of incubation, the concentration of total VFAs in experiment M1 decreased slightly from 2100 to 1950 mg/L and then rapidly decreased to 720 mg/L on day 8. After that, it gradually decreased to a negligible concentration of 70 mg/L, indicating that supplementing suitable amount of fly ash in the first stage could facilitate the conversion of VFAs in the second methanogenic stage. The degradation pattern of VFAs in experiment M2 was almost similar to that in experiment M1. In other words, during the first 6 days, a slight decrease (from 1880 to 1650 mg/L) was observed in the total VFAs concentration, and on day 8, a significant VFAs reduction (from 1650 to 950 mg/L) was occurred, which, however, was smaller than that of experiment M1. Also, in the rest of the methanogenic period, the concentration of total VFAs decreased with a gradual trend and reached 190 mg/L at the end. In the case of experiment M3, which was fed with fly ash-free hydrogenogenic spent medium, a different performance was observed compared to experiments M1 and M2. Furthermore, the concentration of VFAs in M3 increased from 880 to 1460 mg/L during the first 6 days, most likely due to the further conversion of substrate compounds (e.g., proteins) that were incompletely decomposed in the H_2_-producing stage. Afterward, the VFAs were gradually degraded and reached a concentration of 400 mg/L at the end, which was higher than the final concentration of VFAs in the other two methanogenic assays.

As shown in Fig. 8c, the pH in all three CH_4_ production tests gradually increased over time, with the difference that the values recorded in experiments M1 (7.0˗8.1) and M2 (6.3˗7.2), in which the fermentation spent media containing fly ash were used as substrates, were much higher than those in experiment M3 (4.1˗5.0) without fly ash addition. It should also be noted that in experiment M1, the pH remained within the optimal range reported for the function of methanogens (6.5˗8.2) [47] during the entire incubation time. Therefore, all the promising results obtained for experiment M1 demonstrate that the optimization of the first dry H_2_-producing stage by the proposed approach could positively affect the performance of the second stage of CH_4_ production.

### Overall performance of the two-stage biohythane production

The H_2_/(H_2_ + CH_4_) volume ratio is considered a useful indicator for evaluating the performance of the two-stage biohythane production processes [48]. According to the literature, H_2_ contents of 10˗25% (by volume) are desirable to improve combustion efficiency as well as reduce greenhouse gas emissions when using biohythane as a vehicle fuel [1]. In the present study, the H_2_ contents in the biohythane obtained from the H_2_ and CH_4_ production experiments H(Optimized) + M1, H9 + M2, and H4 + M3 were 19%, 18%, and 9%, respectively (see Table 5). Therefore, in the first and second two-stage AD processes, the H_2_ content was within the suggested optimal range, while in the third one, it was less than the lower limit.

**Table 5:** Performance of the two-stage AD processes for biohythane production in this work

Two-stage AD	H_2_/(H_2_ + CH_4_)	Energy yield (kJ/gVS_added_)				VS removal efficiency (%)		
		1^st^ stage	2^nd^ stage	Total		1^st^ stage	2^nd^ stage	Total
H(Optimized) + M1	0.19	1.2	15.9	17.1		31	46	77
H9 + M2	0.18	1	14.3	15.3		20	45	65
H4 + M3	0.09	0.1	3.8	3.9		5	18	23

The energy yields and VS removal efficiencies of the aforementioned two-stage AD processes are also summarized in Table 5. Energy yield analysis was performed based on higher heating values of H_2_ and CH_4_ under STP conditions. As shown in Table 5, the energy yield in the hydrogenogenic and methanogenic stages varied from 0.1 to 1.2 and from 3.8 to 15.9, respectively. The overall energy yields of H(Optimized) + M1, H9 + M2, and H4 + M3 (excluding energy consumed) were 17.1, 15.3, and 3.9 kJ/gVS_added_, respectively, showing a direct relationship with the amount of biomass fly ash added. Although a fully batch regime was applied to the two-stage AD processes in the present study, the maximum overall energy yield was comparable to those obtained in previous continuous/semi-continuous two-stage configurations treating OFMSW and food waste, both in dry [44, 45] and wet [49–53] conditions.

Higher VS removal efficiencies are usually linked with higher conversion rates of organic matter to biogas. As can be seen in Table 5, the VS removal efficiency in the hydrogenogenic reactors was in the increasing order of H4 (5%) < H9 (20%) < H(Optimized) (31%), indicating that the increase in biomass fly ash dosage enhanced the hydrolysis-acidogenesis of OFMSW. Regarding the methanogenic stage, the obtained values for experiments M1 (46%) and M2 (45%) were very close to each other and higher than that in experiment M3 (18%). The total VS removal efficiencies in the integrated two-stage processes H(Optimized) + M1, H9 + M2, and H4 + M3 were 77%, 65%, and 23%, respectively, in line with the total biogas yields obtained from the two-stage biological systems designed in this work. The highest total VS removal efficiency attained was also comparable to those in similar two-stage processes (i.e., dry H_2_-stage followed by wet CH_4_-stage) using food waste and OFMSW [44, 54].

In view of all the above, it seems that the use of an appropriate amount of biomass fly ash as a cost-effective additive in the solid-state hydrogenogenic stage followed by a methanogenic stage could provide an opportunity for the economical production of biohythane with a favorable composition and further bio-energy recovery from organic waste without the use of commercial alkalis and buffers.

## Conclusions

In this study, the use of Doehlert experimental design enabled the fast and efficient optimization of the solid-state H_2_ production stage supplemented with biomass fly ash in a two-stage AD process for biohythane production from OFMSW. The generated mathematical model fitted well with the experimental data obtained from nine designed batch fermentation tests, indicating a high predictability of the model. By applying the optimum conditions extracted from the Doehlert matrix for the TS content (29.1%) and fly ash dosage (19.2 g/L) in the first stage of the two-stage AD process, the observed H_2_ yield (95 mL/gVS_added_) agreed well with the value predicted by the model (97 mL/gVS_added_). The first solid-state H_2_ fermentation stage augmented with appropriate amount of fly ash also favored the subsequent methanogenic stage, leading to a high CH_4_ yield of 400 mL/gVS_added_ which was about 76% of the theoretical CH_4_ yield. Overall, the proposed approach resulted in a relatively high energy yield (17.1 kJ/gVS_added_) as well as an optimal H_2_ fraction (19% v/v) in the biohythane obtained from the integrated two-stage process, demonstrating its potential for large-scale implementation.

## Data Availability

The datasets used and/or analyzed during the current study are available from the corresponding author on reasonable request.
